# Physiological and Biochemical Markers of the Sex-Specific Sensitivity to Epileptogenic Factors, Delayed Consequences of Seizures and Their Response to Vitamins B1 and B6 in a Rat Model

**DOI:** 10.3390/ph14080737

**Published:** 2021-07-28

**Authors:** Vasily A. Aleshin, Anastasia V. Graf, Artem V. Artiukhov, Alexandra I. Boyko, Alexander L. Ksenofontov, Maria V. Maslova, Isabel Nogués, Martino L. di Salvo, Victoria I. Bunik

**Affiliations:** 1 Faculty of Bioengineering and Bioinformatics, Lomonosov Moscow State University, 119991 Moscow, Russia; aleshin_vasily@mail.ru (V.A.A.); whitelord_1994@mail.ru (A.V.A.); boiko.sash@gmail.com (A.I.B.); 2 A.N. Belozersky Institute of Physicochemical Biology, Lomonosov Moscow State University, 119991 Moscow, Russia; nastjushka@gmail.com (A.V.G.); ksenofon@belozersky.msu.ru (A.L.K.); 3 Faculty of Biology, Lomonosov Moscow State University, 119234 Moscow, Russia; maslova_masha@mail.ru; 4 Faculty of Nano-, Bio-, Informational, Cognitive and Socio-Humanistic Sciences and Technologies at Moscow Institute of Physics and Technology, 123098 Moscow, Russia; 5 Research Institute of Terrestrial Ecosystems, National Research Council, Via Salaria km 29.300, Monterotondo, 00015 Rome, Italy; mariaisabel.noguesgonzalez@cnr.it; 6 Department of Biological Sciences A. Rossi Fanelli, Sapienza University, 00185 Rome, Italy; martino.disalvo@uniroma1.it; 7 Department of Biochemistry, Sechenov University, Trubetskaya, 8, bld. 2, 119991 Moscow, Russia

**Keywords:** amino acid neurotransmitter, glutamate, GABA, pentylenetetrazole, vitamin B1, vitamin B6

## Abstract

The disturbed metabolism of vitamins B1 or B6, which are essential for neurotransmitters homeostasis, may cause seizures. Our study aims at revealing therapeutic potential of vitamins B1 and B6 by estimating the short- and long-term effects of their combined administration with the seizure inductor pentylenetetrazole (PTZ). The PTZ dose dependence of a seizure and its parameters according to modified Racine’s scale, along with delayed physiological and biochemical consequences the next day after the seizure are assessed regarding sexual dimorphism in epilepsy. PTZ sensitivity is stronger in the female than the male rats. The next day after a seizure, sex differences in behavior and brain biochemistry arise. The induced sex differences in anxiety and locomotor activity correspond to the disappearance of sex differences in the brain aspartate and alanine, with appearance of those in glutamate and glutamine. PTZ decreases the brain malate dehydrogenase activity and urea in the males and the phenylalanine in the females. The administration of vitamins B1 and B6 24 h before PTZ delays a seizure in female rats only. This desensitization is not observed at short intervals (0.5–2 h) between the administration of the vitamins and PTZ. With the increasing interval, the pyridoxal kinase (PLK) activity in the female brain decreases, suggesting that the PLK downregulation by vitamins contributes to the desensitization. The delayed effects of vitamins and/or PTZ are mostly sex-specific and interacting. Our findings on the sex differences in sensitivity to epileptogenic factors, action of vitamins B1/B6 and associated biochemical events have medical implications.

## 1. Introduction

Triggered by abnormal brain activity, generally due to a disbalance between neuronal excitation and inhibition, seizures are a hallmark of epilepsy, a common and predominantly polygenic disease. In more than a half of the cases, the cause of epilepsy remains unknown. Some epilepsies, called vitamin-dependent, belong to rare genetic disorders caused by mutations in the genes involved in the metabolism of vitamins B1 and B6 [1,2,3,4,5]. Normalization of the organismal vitamin status in these types of epilepsy has a therapeutic effect, eliminating seizures that cannot be treated with known anti-seizure medications. Vitamins B1 and B6 may also contribute to other non-monogenic types of epilepsy. Although the role of vitamins in the pathology of epilepsy remains poorly understood, a number of enzymes participating in the metabolism of glutamate and GABA are altered in epilepsy. Those include not only the known targets of vitamin B6 (transaminases and decarboxylases), but also the enzymes (e.g., glutamate dehydrogenase (GDH), malate dehydrogenase (MDH), glutamine synthetase (GS) and others) whose function may be regulated by vitamin B1 beyond the coenzyme action of this vitamin on the thiamine-diphosphate-dependent enzymes, such as the dehydrogenases of 2-oxo acids [6,7,8,9,10,11,12]. Subclinical hypovitaminosis of B1 or B6 may arise in patients treated with widely used medications, including anti-seizure medications [13,14,15,16], potentially facilitating the development of non-monogenic epilepsies. Hence, upon dysfunction in the vitamin metabolism, either acquired or caused by genetic defects, the administration of vitamins B1 and/or B6 may mitigate epileptic seizures and their long-standing consequences. This work aims to characterize potential therapeutic effect of the administration of these vitamins in a rat model of epilepsy, as well as effects of a seizure on the brain central metabolism dependent on vitamins B1 and B6.

Our study takes into account physiological observations on sexual dimorphism in epilepsy [17]. To induce a seizure, we employ a widely used model of pentylenetetrazole (PTZ) administration. Binding to the tert-butylbicyclophosphorothionate site of the GABA-A receptor chloride channel, PTZ causes disbalance between the excitatory and inhibitory pathways of neurotransmission [18,19,20,21]. Physiological manifestations are analyzed by characterizing the severity of a seizure and assessing its delayed effect on behavioral and ECG parameters. To study the potential therapeutic effects of high doses of vitamins, the administration of vitamins B1 and B6 is combined, in view of the B1-dependent regulation of B6 metabolism in animals [11]. The administered doses of the vitamins are equivalent to those known from medical applications in patients. To characterize the molecular mechanisms and pathways involved, the physiological assessment is combined with the analysis of the consequences of a seizure for the brain biochemistry. In this part, the vitamin-dependent enzymes crucial for the brain metabolism of glutamate and GABA [1,2,22,23,24,25,26] are assayed, and the brain amino acid profiles [23,24] are quantified. As a result, we reveal sex differences in the sensitivity to PTZ administration, the biochemical markers of such differences and a dual sex-specific effect of the administration of vitamins B1 and B6 on a seizure associated with the regulation of the brain PLK activity.

## 2. Results

### 2.1. Sex Differences in the Susceptibility of Animals to the Seizure-Inducer PTZ and to the Delayed Consequences of a Seizure

**Figure 1:**
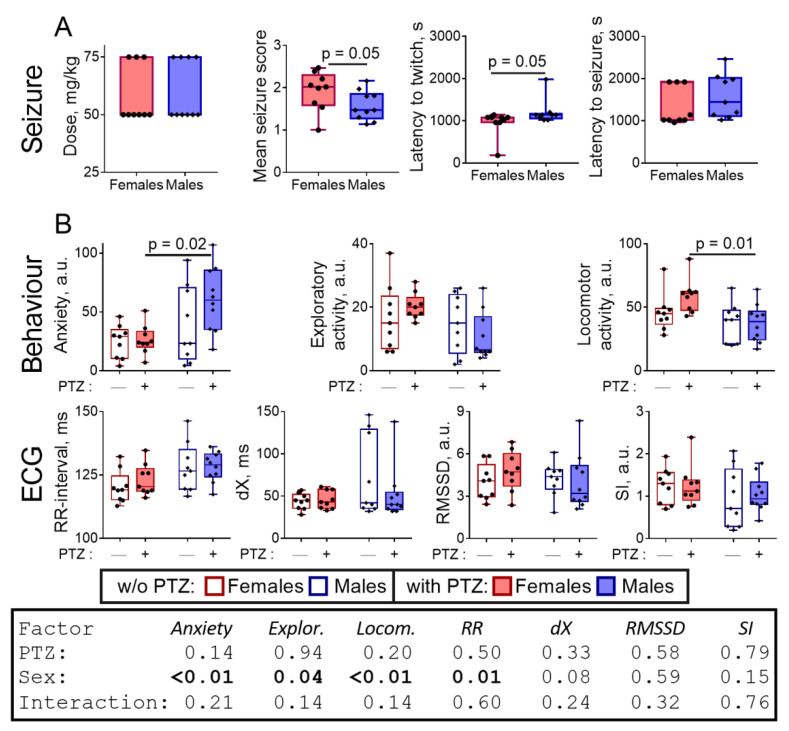
Sex-dependent parameters of the PTZ-induced seizure (**A**) and its delayed physiological effects (**B**) compared to the control animals. The assayed parameters are indicated on the Y axes. (**A**)—“Dose” refers to the total PTZ dose received by an animal for the seizure induction; “Mean seizure score” is determined during the 45 min of the observation; “Latency to twitch” defines the time to the first myoclonic twitch (three points by the modified Racine scale, see “Materials and methods”); “Latency to seizure” defines the time to the first tonic seizure (four points by the Racine scale, see “Materials and methods”). (**B**)—Behavioral and ECG parameters of the rats the next day after a seizure compared to the control groups. The parameters of anxiety, exploratory activity and locomotor activity are obtained from the “open field” test as described in “Materials and methods”. The ECG parameters include the length of the RR interval, the heart variability rate dX, the RMSSD and stress (SI) indices, estimated as described in “Materials and methods”. *p*-values are determined using the Mann–Whitney test (**A**) and two-way ANOVA with Tukey’s post hoc test (**B**) (see Section 4).

The scheme of administration of pentylenetetrazole (PTZ) has been selected to minimize mortality, simultaneously achieving a strong manifestation of the clonic and tonic seizures according to the Racine scale modified for assessment of the PTZ-induced seizure (see Section 4). Accordingly, the PTZ dose received by individual animals varies depending on their individual susceptibility (Supplementary Figure S1). Sensitivity of the male and female rats to the seizure inducer is characterized by an average PTZ dose and the pathophysiological parameters of the seizure development, shown in Figure 1A. The average PTZ dose is practically the same for the female and male rats: 58 ± 4 vs. 60 ± 4 mg/kg, respectively (Figure 1A). However, the mean seizure score is significantly (Mann–Whitney test, *p* = 0.05) lower in the male rats compared to the female ones (Figure 1A). In addition, male rats experience convulsive twitching app. 4 min later (Mann–Whitney test, *p* = 0.05), compared to the female rats (Figure 1A). Thus, the obtained results indicate that female rats are more sensitive to the seizure induction by PTZ than male rats.

The next day after a seizure, the sex differences in anxiety (Tukey’s multiple comparisons test, *p* = 0.02, ANOVA “sex” factor *p* < 0.01; F(1,33) = 10.08), mainly manifested in freezing time (Tukey’s multiple comparisons test, *p* = 0.01, Supplementary Figure S2, ANOVA “sex” factor *p* < 0.01; F(1,33) = 8.87) and locomotion (Tukey’s multiple comparisons test, *p* = 0.01, ANOVA “sex” factor *p* < 0.01; F(1,33) = 9.18), arise that are absent in the control groups (Figure 1B). Obviously, significant sex differences in anxiety and locomotion after a seizure represent a cumulative effect of minor and statistically insignificant shifts exhibited by each of the sexes in response to PTZ. This view is confirmed by the two-way ANOVA pointing to the statistical significance of the factor of sex in the PTZ-induced changes (Figure 1B). In addition to the behavioral parameters, the potential cardiological risks of a seizure [27,28,29] may be estimated by ECG. As seen from Figure 1B, the ECG assay does not reveal significant changes in the rats after the seizure.

**Figure 2:**
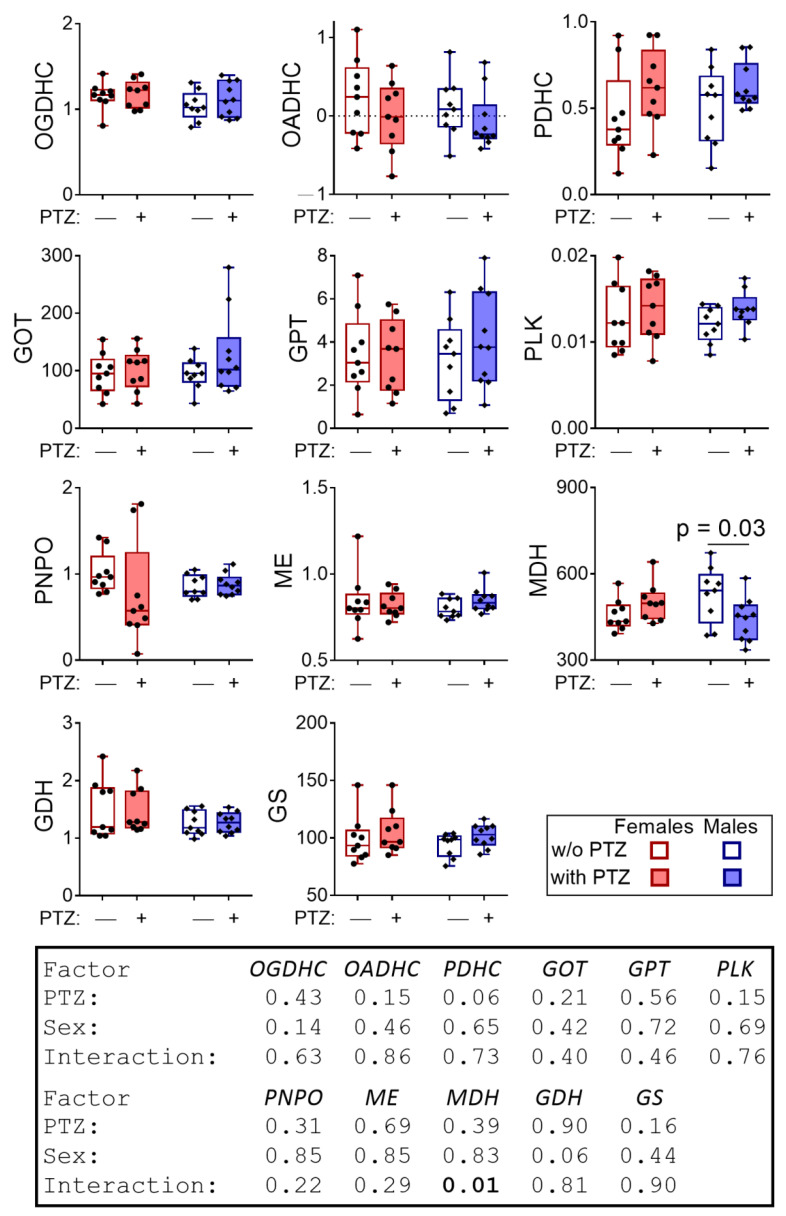
Delayed effect of the PTZ-induced seizure on the activities of cerebral cortex enzymes in the male and female rats compared to the control groups. The activities are presented as micromole/min per g of tissue, with the exception for PNPO, whose activity is in nanomole/min per g of tissue. *p*-values are determined using two-way ANOVA with Tukey’s post hoc test (see Section 4).

The analysis of the biochemical markers in the cerebral cortex of the rats the next day after a seizure did not reveal a significant effect of PTZ on the activity of the tested enzymes of the brains’ neurotransmitter metabolism, with the exception of a decrease in the MDH activity (Tukey’s multiple comparisons test, *p* = 0.03) in males (Figure 2). Significant (*p* = 0.01, F(1,33) = 7.16) interaction of PTZ and sex regarding the MDH activity is revealed by two-way ANOVA, corresponding to the opposite directions of PTZ’s effects on MDH activity in the male and female rats (Figure 2).

**Figure 3:**
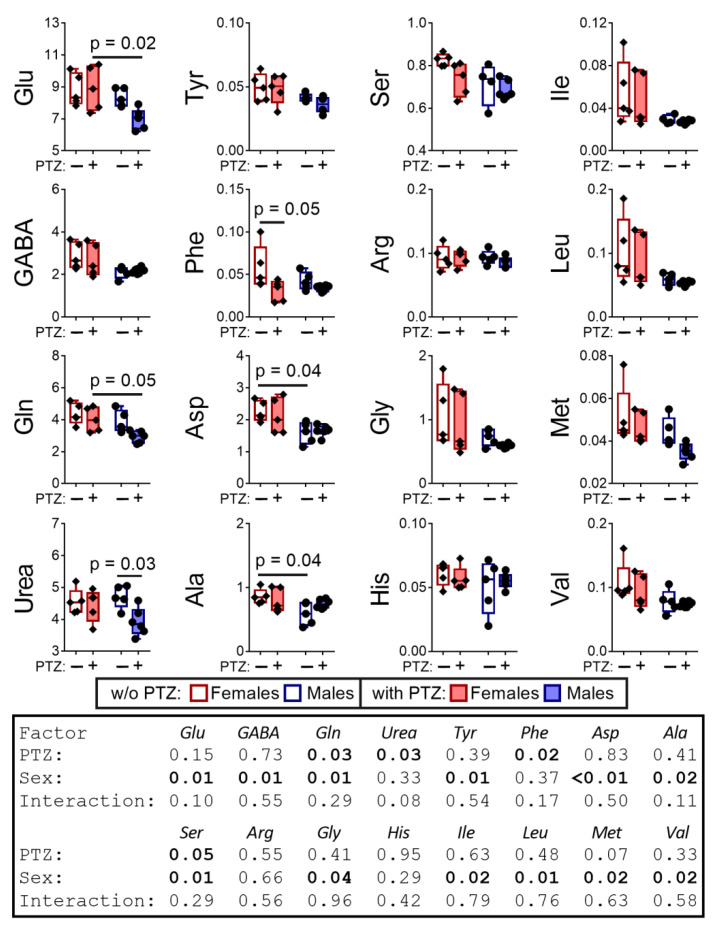
The delayed effect of the PTZ-induced seizures on the levels of amino acids and urea in the cerebral cortex of the male and female rats compared to the control groups. The content of free amino acids and urea is in micromoles per g of tissue. *p*-values are determined using two-way ANOVA with Tukey’s post hoc test (see Section 4).

Of note, the enzymatic reaction rates in vivo depend on the local concentrations of metabolites, which are far from the saturating concentrations used for assays of maximal reaction rates of enzymes in vitro. Therefore, the in vivo levels of metabolites are a better measure of the biological impact of the minor differences in the enzymatic activities, determined in vitro (Figure 2). Indeed, the analysis of the levels of free amino acids in the brain reveals multiple sex-dependent PTZ effects on the metabolites. As seen from Figure 3, PTZ eliminates the pre-existing sex differences in the levels of aspartate and alanine, inducing those in the levels of glutamate and glutamine. The sex-specific effects of PTZ, such as decreases in urea in the brain of male rats, and phenylalanine in the brain of female rats, are less abundant. Accordingly, the two-way ANOVA indicates that the sex factor is essential for 12 out of 15 estimated metabolites, while PTZ is essential for only 4 out of 15 metabolites (Figure 3).

In summary, both the sex differences in the levels of free amino acids in the rat brain and the amino acid levels themselves respond to the action of PTZ. The sex-specific responses of the brain biochemical parameters (Figure 2 and Figure 3) correspond to the different sensitivity of the sexes to PTZ (Figure 1A) and the post-seizure induction of behavioral differences between the sexes (Figure 1B). In particular, the PTZ-induced changes in the sexually dimorphic pattern of the brain amino acids (Figure 3) correspond well to the PTZ-induced sex differences in behavior (Figure 1B) and MDH activity (Figure 2).

### 2.2. Sex-Dependent Effects of Combined Administration of Vitamins B1 and B6 on the Severity of a Seizure and Its Delayed Consequences

**Figure 4:**
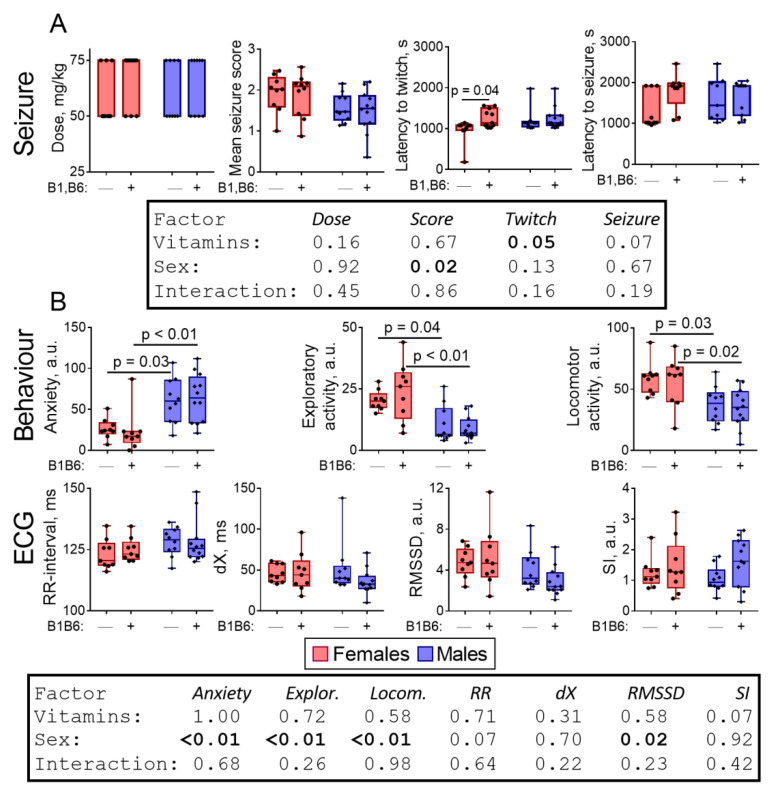
Sex-dependent effects of the administration of vitamins B1 and B6 on the parameters of the PTZ-induced seizure (**A**) and its delayed physiological effects (**B**). The assayed parameters are indicated on the Y axes and described in more details in Figure 1 and “Materials and methods”. *p*-values are determined using two-way ANOVA with Tukey’s post hoc test (see Section 4).

In females, the combined administration of vitamins B1 and B6 24 h before PTZ increases the time until the twitching (Tukey’s multiple comparisons test, *p* = 0.04, ANOVA “Vitamins” factor *p* = 0.05; F(1,37) = 4.27), but in males, the parameters of the seizures remain unaffected (Figure 4A). Thus, the higher the animal susceptibility to the seizure-inducing action of PTZ (Figure 1A), the greater the animal sensitivity to the protective action of vitamins against such action (Figure 4A).

As for the delayed effect of the PTZ-induced seizure on behavior, vitamins slightly enhance the sex differences arising after the seizure (Figure 4B, Supplementary Figure S3). This is observed from an increase in significance of the behavioral differences between the male and female rats after the administration of both PTZ and vitamins (*p* ≤ 0.02) compared to similar groups with no administered vitamins (Tukey’s multiple comparisons test, *p* ≥ 0.03) (Figure 4B). Together, these sex differences manifest in ANOVA “sex” factor significances for anxiety (*p* < 0.01; F(1,36) = 22.25), exploratory activity (*p* < 0.01; F(1,36) = 27.29) and locomotor activity (*p* < 0.01; F(1,36) = 17.44). No significant effect of the vitamins on the ECG parameters is observed (Figure 4B).

**Figure 5:**
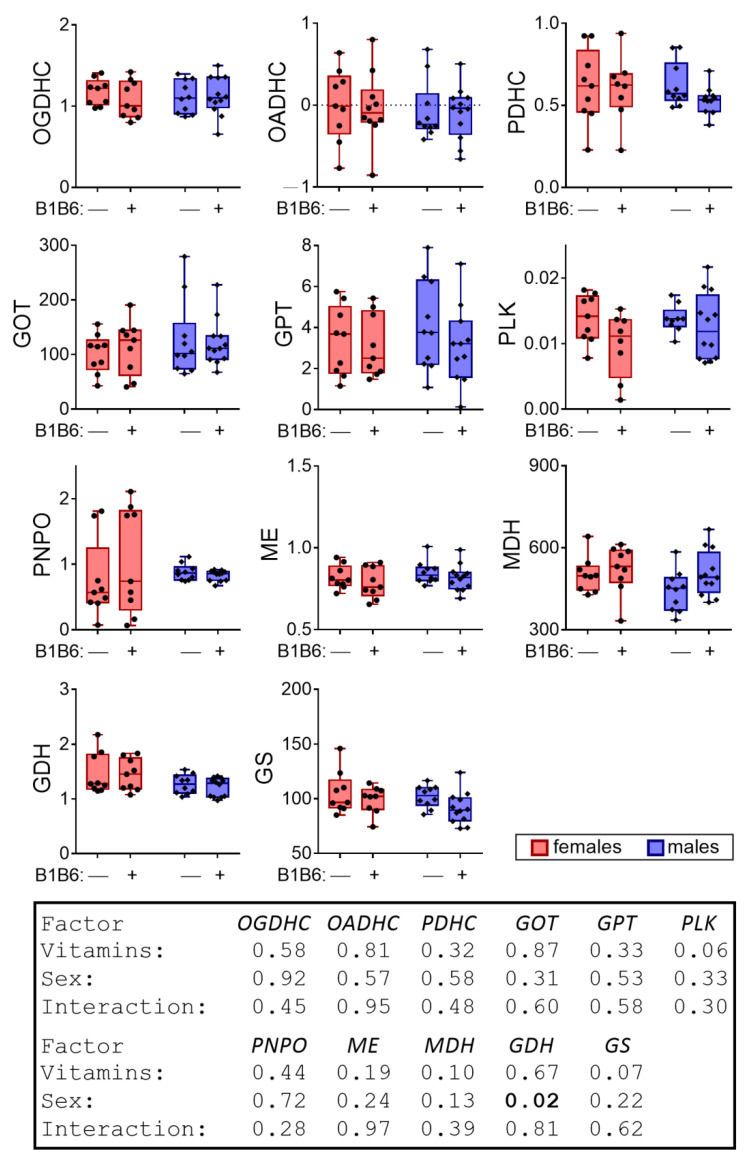
Effects of the administration of vitamins B1 and B6 on the activities of the cerebral cortex enzymes in the male and female rats the next day after the PTZ-induced seizure. The activities of enzymes are expressed in micromole/min per g of tissue, with the exception of the PNPO activity, expressed in nanomole/min per g of tissue. The statistical analysis of the data was performed by the two-way ANOVA with Tukey’s post hoc test (see Section 4).

Biochemical analysis of the rat brain shows no pronounced changes upon the combined action of PTZ and vitamins in comparison with the action of PTZ alone. Two-way ANOVA analysis of these animal groups reveals only the sex-dependent difference (*p* = 0.02, F(1,36) = 6.46) in GDH activity (Figure 5).

**Figure 6:**
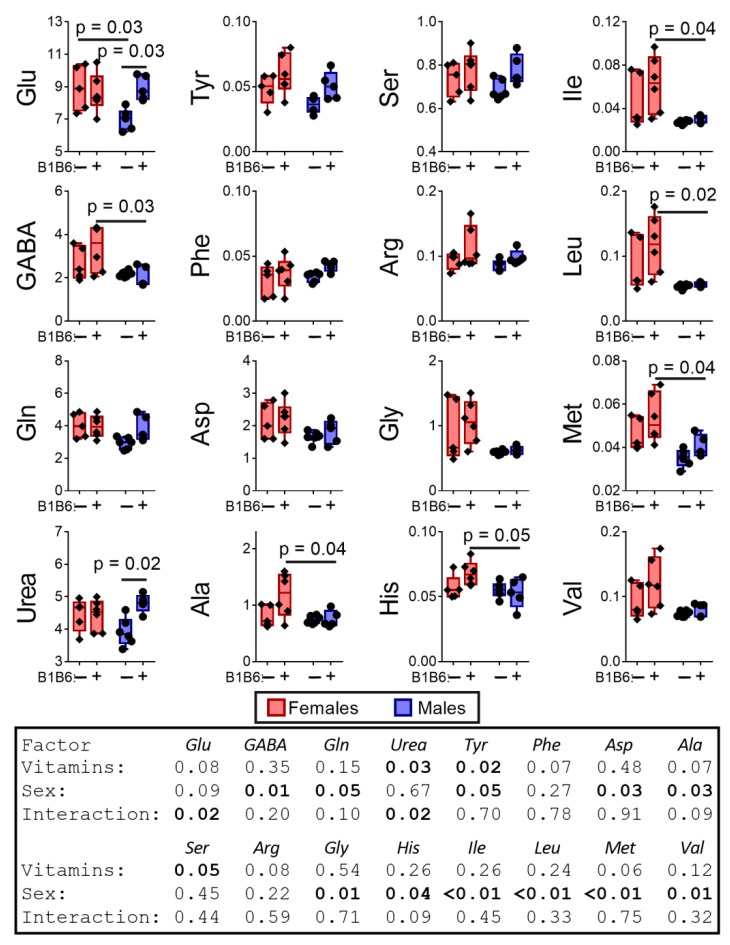
Effects of the administration of vitamins B1 and B6 to the PTZ-treated animals on the levels of amino acids and urea in the cerebral cortex of the male and female rats the next day after the PTZ-induced seizure. The metabolites are indicated on the Y axes, their content is expressed in micromoles per g of tissue. The statistical analysis of the data was performed by the two-way ANOVA with Tukey’s post hoc test (see Section 4).

Similar to the effects of PTZ alone, upon the combined administration of the vitamins and PTZ, the brain metabolite levels undergo much more pronounced changes than the brain enzymatic activities do. When comparing the effects of vitamins in the PTZ-treated animals (Figure 6) to the action of PTZ on the control animals (Figure 3), the partial reversal of the PTZ effects by vitamins is remarkable. The PTZ-induced or abrogated sex differences in the levels of cerebral glutamate, glutamine and alanine (Figure 3) are returned to the control pattern in the vitamin-supplemented animals exposed to PTZ (Figure 6). This is accompanied by increases in the levels of glutamate and urea in the vitamin-supplemented PTZ males vs. those without vitamins (Figure 6), the effects being opposite to those of PTZ (Figure 3). However, the PTZ-perturbed sex relationship in the level of aspartate (Figure 3) is not normalized by the vitamins administration (Figure 6). In the female brain, the vitamins do not return the phenylalanine level (Figure 6) to its control value, which is decreased by PTZ (Figure 3). Moreover, the combined administration of vitamins and PTZ induces additional sex differences in the levels of GABA, histidine, methionine and the branched-chain amino acids isoleucine and leucine (Figure 6), which are not statistically significant in either the control or the PTZ-treated rats (Figure 3). Thus, the administration of vitamins B1 and B6 24 h before PTZ restores sex-dependent levels of some amino acids, simultaneously enhancing the sex differences in the levels of other amino acids. While the former effect of the vitamins administration to the PTZ-treated animals is only observed in the biochemical parameters, the vitamin-induced enhancement of sex differences in the brain levels of GABA, histidine, methionine, isoleucine and leucine corresponds well to the enhancement of sex differences in physiological parameters (Figure 4B).

### 2.3. Effects of the Administration of Vitamins B1 and B6 to the Animals Not Exposed to PTZ

**Figure 7:**
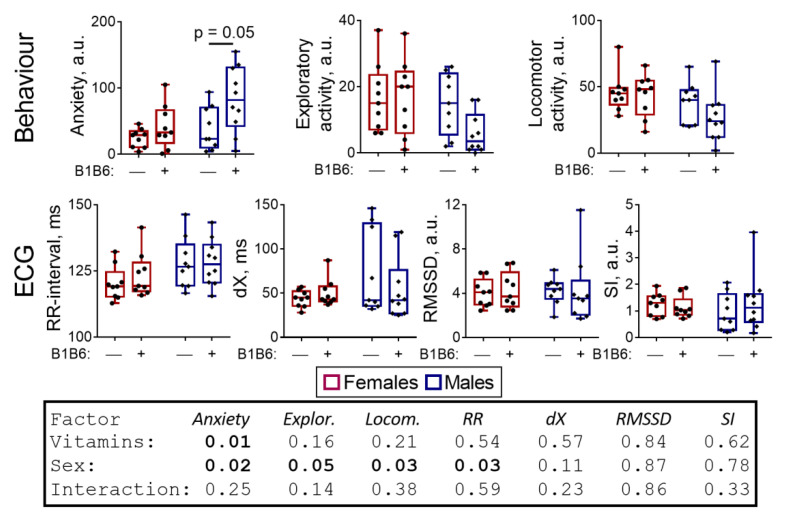
Physiological effects of the administration of vitamins B1 and B6 to the control rats. The studied parameters are indicated on Y axes and described in detail in Figure 1 and “Materials and methods”. The statistical analysis of the data was performed by the two-way ANOVA with Tukey’s post hoc test (see Section 4).

In view of the revealed enhancement by the vitamins of the delayed PTZ effects, shown above, the male and female rats not exposed to PTZ, i.e., the control groups, have been treated with the vitamins according to the same scheme as the PTZ-treated animals (Supplementary Figure S1). As seen from Figure 7, the female rats are not affected by the vitamins administration, but the male rats demonstrate an increase in anxiety (Tukey’s multiple comparisons test, *p* = 0.05; the “vitamins” factor of the two-way ANOVA *p* = 0.01; F(1,33) = 6.97) (Figure 7), mostly contributed by an increase in freezing time (Tukey’s multiple comparisons test, *p* = 0.02; the “vitamins” factor of the two-way ANOVA *p* = 0.02; F(1,33) = 8.87, Supplementary Figure S4). The administration of the vitamins also leads to significant sex differences in the central activity, freezing time, grooming acts and defecation acts (Supplementary Figure S4). No significant effect on the ECG parameters is observed. Two-way ANOVA reveals the importance of the sex factor in the three behavioral parameters and the RR interval of ECG (Figure 7), which corresponds well to the sex-dependent differences, observed in Figure 1B.

**Figure 8:**
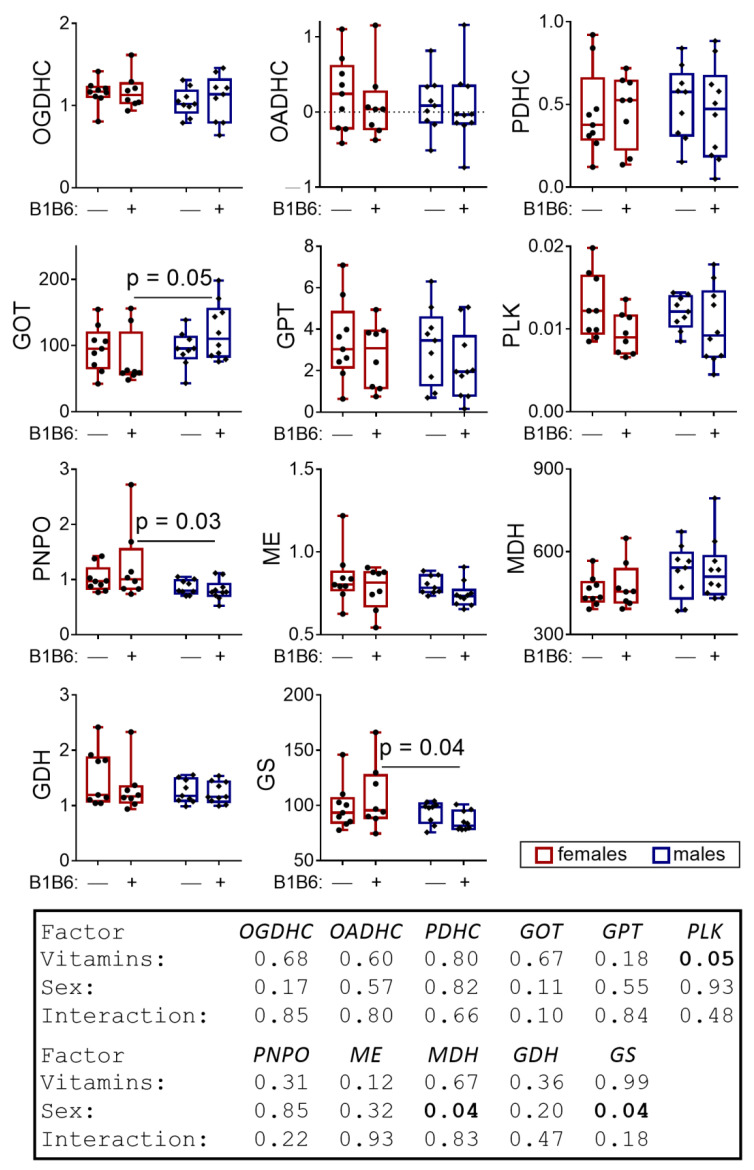
Effects of the administration of vitamins of B1 and B6 to the control rats on the enzymatic activities in the rat brains. The activities are expressed in micromole/min per g of tissue, with the exception for the PNPO activity, expressed in nanomole/min per g of tissue. The statistical analysis of the data was performed by the two-way ANOVA with Tukey’s post hoc test (see Section 4).

**Figure 9:**
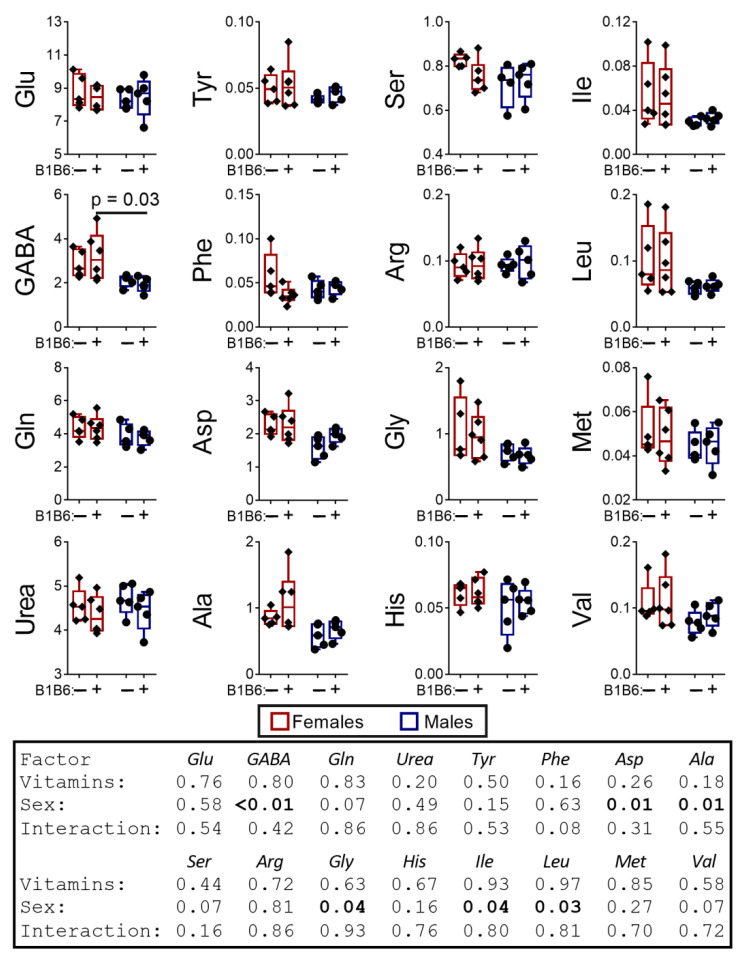
Effects of the administration of vitamins of B1 and B6 to the control rats on the levels of amino acids and urea in the rat brain. The content of free amino acids and urea is shown in micromoles per g of tissue. The statistical analysis of the data was performed by the two-way ANOVA with Tukey’s post hoc test (see Section 4).

The administration of vitamins to the control rats induces significant sex-dependent effects on the levels of the enzymatic activities (Figure 8). That is, the administration of vitamins induces the sexual dimorphism in the levels of GOT, PNPO and GS (Figure 8). A significant (*p* = 0.05, F(1,32) = 4.33) effect of the vitamins on the PLK activity regardless of sex is also revealed by two-way ANOVA (Figure 8). The comparison of the vitamins’ effects on the amino acid profiles in the control rats (Figure 9) with those after PTZ (Figure 6) indicate that the vitamins similarly affect the sex relationships in the level of GABA in both the PTZ-treated (Figure 6) and the control (Figure 9) animals. However, in the PTZ-treated animals, this effect of the vitamins antagonizes the opposite effect induced by PTZ (Figure 3). No other significant effects of vitamins on the amino acids levels are observed in the brain of control rats (Figure 9).

Accordingly, most of the sex effects induced by the combined action of the vitamins and PTZ on the brain amino acid levels (Figure 6) are not observed upon the action of each of the factors (Figure 3 and Figure 9), thus arising due to synergistic action of vitamins and PTZ upon their combined administration.

### 2.4. Interval between Administration of Vitamins B1/B6 and PTZ Influences the PTZ-Induced Seizure and Its Consequences in Females

**Figure 10:**
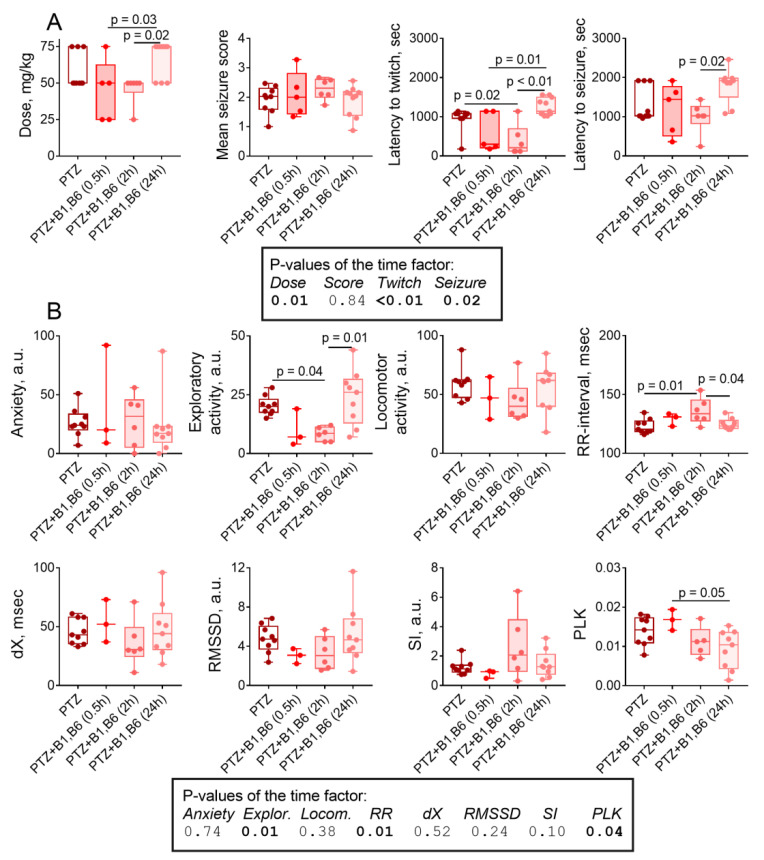
Dependence of pathophysiological parameters of the PTZ-induced seizure (**A**) and its delayed physiological and biochemical effects (**B**) in female rats on the interval between the administration of vitamins B1/B6 and PTZ. The assayed parameters are indicated on the Y axes and described in “Materials and methods”. The activity of the female brain PLK (micromole/min per g of tissue) is used as an indicator of biochemical changes. The statistical analysis of the data was performed by the one-way ANOVA with Tukey’s post hoc test (see Section 4).

In view of a higher sensitivity of female rats to both PTZ and the anti-seizure action of the vitamins (Figure 1 and Figure 4), the time-dependence of the administration of vitamins B1 and B6 before PTZ has been studied in female rats, comparing the series of animals with the “long-term” (24 h) and “short-term” (2 or 0.5 h) intervals between the vitamins and PTZ administrations (Supplementary Figure S1). In contrast to the anti-seizure action of the vitamins administered 24 h before PTZ, the administration of vitamins 0.5 or 2 h before PTZ enhances the severity of the seizures. The animal group where vitamins have been administered 0.5 h before PTZ has demonstrated an increased rate of deadly seizures in contrast to 100% survival in all other groups (see Section 4.4). Stronger seizures in these rats are confirmed by a lower doze of PTZ causing seizures and an earlier onset of twitching and tonic seizures, compared to the rats, receiving vitamins 24 h before PTZ (Figure 10A). The time dependence of the action of vitamins in females confirms an essential difference in the effects of vitamins administration at a short (2 h) or long (24 h) time interval before PTZ, underlying the opposite physiological effect of the vitamins in these cases (Figure 10A). The vitamins administered 0.5–2 h before PTZ, sensitize female rats to the action of PTZ, while the vitamins administered 24 h before PTZ has an anti-seizure effect.

The delayed consequences of a seizure in females further confirm the opposite effects of the vitamins administration 2 and 24 h before PTZ. This is observed as a significant drop in exploratory activity (mainly because of the effect on the central activity in the “open field”, Supplementary Figure S5) and an increase in the RR interval of ECG in the rats with the short-term vitamins administration compared to the rats which received vitamins 24 h before PTZ or do not receive vitamins at all (Figure 10B). The low level of exploratory activity in the groups of rats receiving the vitamins 0.5 or 2 h before PTZ (Figure 10B) is consistent with the parameters of seizures, indicating that administration of vitamins at these time points sensitizes the female rats to PTZ. Overall, the physiological indicators of the delayed effects of a seizure are in good agreement with the pathophysiological parameters of the seizure itself, testifying to the facilitation of a seizure after the short-term administration of vitamins prior to PTZ and to a protective effect of the vitamins administered 24 h before a seizure (Figure 10A).

Remarkably, the administration of vitamins 24 h before PTZ results in significantly (Tukey’s multiple comparisons test, *p* = 0.05; ANOVA *p* = 0.04, F(3,22) = 3.36) lower PLK activity compared to the administration of the vitamins 0.5 h before PTZ (Figure 10B). Thus, the PLK decrease during the long interval between the administration of vitamins and PTZ is associated with the protective effect of vitamins against the PTZ-induced seizure.

## 3. Discussion

In humans, gender differences in epilepsy and underlying molecular mechanisms remain a well-known challenge for therapeutic approaches [17,30]. The accumulating data point to significant interactions between the hormonal status and other factors in epilepsy [17], which should be taken into account to improve the therapy of epilepsy. Not only do steroid hormones and sexually dimorphic neural networks support differential susceptibility of males and females to specific epileptogenic factors, but neurosteroids do as well [31,32,33]. The most studied neurosteroids allotetrahydrodeoxycorticosterone and allopregnanolone modulate the function of the GABA_A_ receptor, thus controlling the neural network excitability, which is perturbed in epileptic seizures [31]. The estrous cycle in sexually mature females represents an associated challenge, requiring more specific studies in females. For instance, the term catamenial epilepsy refers to female seizures with periodic occurrence depending on their menstrual cycle [32]. Mostly, estrous-cycle-related differences in physiological parameters and stress reactions are observed between the luteal (metestrus/diestrus) and follicular (proestrus/estrus) phases, which strongly differ in the levels of sex hormones [34,35,36,37,38,39]. In our model study, only the female rats in the luteal phase are included, as they represent the majority under the employed experimental conditions. Similar to other studies regarding different parameters [34,35,36,37,38,39,40], we did not observe significant differences between the diestrus and metestrus rats, owing to which they were pooled together. Based on the strongly different hormonal status of the female rats in follicular phase, compared to those in luteal phase [34,35,36,37,38,39], it is probable that more sexual differences could be revealed between the males and females in follicular phase than were observed in our current work comparing the male rats with the females in luteal phase. This important question requires further studies.

While most of the clinical and experimental studies in epilepsy focus on seizures themselves, the delayed consequences of seizures are not at all less important, as the accumulation of even minor changes in the brain homeostasis may lead to a vicious cycle deteriorating the patients state with time. Employing the PTZ-induced model of seizures, we have studied not only the parameters of seizures, but also their physiological consequences and biochemical basis the day after. By comparison of the seizures in the male and female rats, we show that the different reaction of the sexes to the seizure-inducing and protective factors is not always obvious as significant effects in each of the group but may be manifested as an induction of sex differences, or, contrarily, as the disappearance of such differences. Both events obviously result from minor sex-specific changes in opposite directions, which do not reach statistical significance in each sex. The comparison of the males and females is therefore more powerful to detect the sex-specific effects. Using this comparative analysis, we have characterized induction or abrogation by PTZ and/or vitamins of sex differences, absent or pre-existing, correspondingly, in the non-affected state. The data obtained in this work show that such cumulative effects are observed in physiological and/or biochemical parameters, being much more pronounced than the specific actions of PTZ and/or vitamins in the female or male rats. For instance, in our study of the action of vitamins on the PTZ-treated animals (Figure 6), eight statistically significant (*p* ≤ 0.05 by Tukey’s multiple comparisons test) sex differences in the amino acid profiles are affected by the vitamins (of those, six arise and two disappear). However, the statistically significant effects of the vitamins on each sex include only two changes in the male rats and none in the female rats.

The seizure-induced sexual differences correspond to the differential susceptibility of the sexes to the delayed consequences of a seizure (Figure 4B). Moreover, the seizure-induced sexual dimorphism in the level of glutamate suggests a sex-dependent modification of further reactivity to the seizure inducer. In this regard, the PTZ-induced sexual dimorphism in the brain methionine level also deserves attention, as methionine is a precursor of a pro-convulsant homocysteine [41]. Homocysteine levels are higher in the plasma of males compared to females [42], but their increase is more dangerous to the female brain than the male brain [43]. In the animals without exposure to PTZ, there is no difference in the brain methionine level between the males and females (Figure 9). However, the difference tends to arise after PTZ treatment and becomes statistically significant after the combined treatment with PTZ and vitamins (Figure 6). The resulting levels of methionine are higher in females than males, pointing to sex-dependent changes in metabolic pathways involving homocysteine. Changes in these pathways are known to occur after recurrent seizures and antiepileptic drugs, which increase plasma homocysteine levels [41].

All together our experiments reveal multiple sex-specific responses to seizures and therapeutic approaches. Such responses are revealed in the action of PTZ, the delayed consequences of a seizure and the action of vitamins B1 and B6. Changes in the brain amino acids provide very sensitive indicators of the cumulative physiological responses, while the in vitro assays of the brain enzymes reveal the affected parts of metabolic networks. Compared to the control rats, PTZ action decreases the brain MDH only in the male rats. Such subtle difference in the functional expression of one out of eleven tested brain enzymes of the central metabolism (Figure 2) is accompanied by multiple changes in the brain amino acids, including glutamate (Figure 3), which is of utmost significance in epilepsy.

Thus, delayed consequences of a seizure involve changes in the central metabolism of the brain. The dependence of such metabolism on vitamins may result in so-called metabolic epilepsies, often positively responding to administration of vitamins [44]. While deficiencies in vitamins B1 and B6 due to malnutrition have not been considered an issue in developed countries for a long time, these states currently occur more and more often as a result of intensified medical treatments, such as the administration of diuretics, metformin, antibiotics, chemotherapy and bariatric surgery [13,16,26,45]. Regarding vitamins B1 and B6, seizures are known to arise due to mutations in proteins of their transport and metabolic transformations, affecting the physiological function of the vitamins [46,47,48,49]. In the diagnosed cases of neonatal epilepsies originating from the pathogenic mutations, the urgent administration of the required vitamin is essential to prevent not only seizures, but also the following mental retardation [48,49,50]. It should be noted, however, that less pathogenic mutations in the proteins of vitamin metabolism may be compensated under normal physiological conditions, yet their pathogenicity may increase under stress and/or medical interventions. This feature may underlie interindividual variations in developing the vitamins deficiencies promoted by pathophysiological conditions. In such cases, pharmacological doses/forms of vitamins may be a valuable therapeutic option. However, unlike vitamin B1, whose high pharmacological doses do not usually have damaging effects [13], high doses of vitamin B6 are neurotoxic [47] and may induce seizures [51]. Therefore, in view of our finding of the mutual regulation of metabolism of vitamins B6 and B1 [11], the anti-seizure action of the combined administration of both vitamins is studied in this work. Nevertheless, a potentiating effect of the combined administration of vitamins B1 and B6 on the PTZ-induced seizures is observed in female rats upon the vitamins’ administration shortly before PTZ (Figure 10). Only in this animal group have the deadly seizures occurred (see Section 4.4). In the control male rats, the vitamins increase anxiety (Figure 7). The negative effects of the combined administration of B1 and B6 may be ascribed to the action of vitamin B6 in the form of pyridoxal-5′-phosphate, which are known to induce seizures upon intracerebroventricular administration in rats [51]. Such a view is supported by the transformation of the seizures-sensitizing effect of the vitamins administration into a protective one along with downregulation of the brain PLK which synthesizes pyridoxal-5′-phosphate from pyridoxal (vitamin B6) (Figure 10), and increased sex differences in the B6-dependent enzymes GOT and PNPO after the vitamins administration to the control animals (Figure 8). The protective effects of the vitamins introduced 24 h before PTZ also include the vitamins-induced reversal of the PTZ effects on the sex differences in glutamate, glutamine and alanine. As a result, our data reveal molecular mechanisms of therapeutic action of vitamins in combating the delayed consequences of seizures, that warrant further studies.

## 4. Materials and Methods

Reagents from the following manufacturers were used: thiamine—Serva Electrophoresis GmbH, Heidelberg, Germany; pyridoxal—PanReac AppliChem, Barselona, Spain; glycerol—Biomedicals, LLC, Santa Ana, CA, USA; pentylenetetrazole (PTZ), protease inhibitors, buffers and other reagents for the analysis of enzyme activities—Sigma-Aldrich, St. Louis, MO, USA. Pyridoxine-5′-phosphate was synthesized from pyridoxal-5′-phosphate (Sigma-Aldrich, St. Louis, MO, USA) as described previously [52]. The solutions were prepared in Milli-Q deionized water, the salts used were of the highest purity available.

### 4.1. Animals

The studies were carried out in Wistar rats obtained from the Russian Federation State Research Center Institute of Biomedical Problems RAS (IBMP)—both males and females, weighing 250 to 350 g. The animals were kept under standard conditions in cages with free access to water and food and a light/dark cycle of 12/12 h (the light turned on at 9 a.m.). The adaptation period to the husbandry conditions was two weeks. The age of the animals at the time of the study was 2.5–3.0 months. According to the vaginal smears taken an hour before the start of the experiment [53], the female rats were “metestrus” or “diestrus”. Major contributions of females with these stages of estrous cycle, characterized by low levels and minimal fluctuations of sex hormones [54,55], to the experimental animal sample was presumably due to the prolonged duration of these stages (together up to 78 h, i.e., more than 75% of the total duration of the estrous cycle). We did not see any significant differences between the metestrus or diestrus females, hence the animals were combined. Animals were distributed randomly between experimental groups. Animals were killed by decapitation according to the schemes from Supplementary Figure S1, as usage of anesthetics was incompatible with our study of the brain biochemical changes. A long-standing research has established multiple interferences of different anesthetics with metabolic and signaling pathways [56,57,58,59,60,61,62,63]. In view of strong interactions of anesthetics with the mitochondrial function [56,58,59], neurotransmitter levels [58,61,62,63,64] and action of neuroprotectants [61,64], all studied in our work, euthanasia by decapitation was chosen as the most suitable method for our studies on adult animals, in accordance with existing recommendations [63,64,65]. Given that the other animals are not in the decapitation room, cutting off the head using guillotine (OpenScience, Moscow, Russia) is considered one of the least stressful method to kill the animals [63,64,65,66]. After each use, the guillotine was washed thoroughly with water and ethanol, so that no blood smell could stress another animal. The procedure was done according to recommended protocols [63,64,65,66] as described before [67]. Animal experiments and all the described procedures, including euthanasia by decapitation, were approved by Bioethics Committee of Lomonosov Moscow State University (protocol number 69-o from 9 June 2016).

### 4.2. Pentylenetetrazole Model of Seizures

Convulsions were induced by intraperitoneal administration of PTZ in saline at 25 mg/kg dose. After PTZ administration, the severity of seizures was visually assessed according to the modified Racine scale developed for the employed protocol of PTZ administration [68] (Table 1) for 15 min in individual cages (OpenScience, Moscow, Russia). During the visual assessment, there were no more than 4 rats in individual cages (2 with PTZ and 2 without PTZ), arranged so as to allow observation of each of them. Scores were registered every minute of the observation, and an epileptic seizure of maximum score was noted for the given minute of observation. If the stages 4–5 on the modified Racine scale (tonic or tonic-clonic seizures, Table 1) did not develop within 15 min, PTZ was re-injected at the same dose of 25 mg/kg. The procedure was repeated no more than three times, and the total PTZ dose thus did not exceed 75 mg/kg. The total time of the seizure observation was 45 min (Supplementary Figure S1).

The mean seizure score was calculated as the average score of the severity of an epileptic seizure over the entire observation period from the moment of administration of the first PTZ dose.

### 4.3. Vitamin Administration

Vitamins B1 (thiamine, 100 mg per kg body weight) and B6 (pyridoxal, 100 mg per kg body weight) were administered intraperitoneally 24 h before the first PTZ administration and after the completion of a 45 min follow-up of the PTZ-induced seizures (Supplementary Figure S1). In the study of the time-dependence of the vitamins effects, performed on the female rats only, the separate experimental groups received vitamins at varied intervals before the first PTZ administration, i.e., either 24 or 2 or 0.5 h, and after completion of a 45 min follow-up of the PTZ-induced seizures (Supplementary Figure S1). This vitamin regimen took into account the results of previous studies on the potential protective effect of high doses of vitamins both before [72] and after [24] the exposure to stress, when increased availability of vitamins may provide better stabilization and normalization of the metabolic state, respectively [73]. Control animals received the injections with equivalent volumes of physiological solution (0.9% NaCl). Since the seizure occurred before the second injection of vitamins, only the first injection of vitamins affected the convulsions themselves. Both the first and the second doses of vitamins affected the delayed consequences of a seizure.

A major part (about 70%) of both vitamins is known to be excreted from the body 24 h after the injection [74,75]. However, the injections significantly increase the internal pool of vitamins [76] stored in the liver, where concentrations of thiamine diphosphate and B6 vitamers are higher than in other tissues [14,77].

According to the formula recommended by the US Food and Drug Administration (http://www.fda.gov/downloads/Drugs/GuidanceComplianceRegulatoryInformation/Guidances/ucm078932.pdf; accessed on 19 June 2021), the dose of vitamins B1 and B6 used in this study on rats (100 mg/kg each of the vitamins) corresponds to a dose of 16 mg/kg, or 1 g for an average weight of 60 kg, in humans. No toxic or side effects in humans are known for vitamin B1 at a daily dose of 0.5 g for a month [78]; clinically used injections of vitamin B1 may be up to 0.5 g three times a day [79]. However, high doses of vitamin B6 are neurotoxic in humans [75,80], with 2–6 g of vitamin B6 per day resulting in acute sensory neuropathies [75,81]. Intracerebroventricular administration of pyridoxal-5′-phosphate and its synthetic analogs led to seizures in rats [51]. At the same time, such effects were not reported in patients with various B6-dependent syndromes who received 0.5–1.5 g of vitamin B6 per day [75]. Thus, the dose of vitamins B1 and B6 used in our study are within the interval of the doses clinically used in megavitamin therapy. In addition, taking into account the inhibition by vitamin B1 of PLK [11], which produces the coenzyme form of vitamin B6, the co-administration of vitamins used in this study could reduce the B6 neurotoxicity, potentially linked to production of the coenzyme pyridoxal-5′-phosphate by PLK.

### 4.4. Animal Survival

The data on the total number of animals in our experiments and their survival is summarized in Table 2.

### 4.5. Assessment of Physiological Parameters

To assess the spontaneous behavior of animals in an unfamiliar environment, we used the Open Field test (OpenScience, Moscow, Russia) [82]. Standard assays and interpretations of the open-field data were in accordance with the published papers [83,84,85]. The round arena made of polyvinyl chloride with a diameter of 97 cm, surrounded by a wall of 42 cm height, was used (OpenScience, Moscow, Russia). The bottom of the arena was divided into the two circles equidistant from each other (the diameter of the smaller circle was 23 cm, the diameter of the larger circle was 60 cm). The ring formed by the larger circle and the wall of the field was divided into 12 sectors; the ring formed by the larger and smaller circles was divided into 6 sectors. The animals were put in the center and tested in complete silence under the light of a 15 W red lamp for three minutes, assessing the following indicators:

1. locomotor activity—the number of segments passed;
2. rearing acts—the number of stands on the hind limbs;
3. central activity—the number of the intersections of the small and central circles plus the number of movements from the walls intersecting the larger circle. In animals that did not move from the center (freezing), this indicator was equal to 0;
4. total freezing time—time when the active behavior was lacking in all the sectors;
5. grooming time;
6. the number of acts of grooming;
7. the number of defecation acts.

In the graphs of Results, the parameters were partly combined as cumulative indices for exploratory activity and anxiety. The exploratory activity summarized the rearing acts and central activity; the anxiety summarized freezing and grooming times with the number of grooming and defecation acts. All parameters used to calculate the cumulative indicators were assigned arbitrary units, such as, e.g., 1 s of freezing = 1 a.u.; 1 act of defecation = 1 a.u., etc. The employed combinations of parameters to characterize anxiety and exploratory activities were based on the studies showing unidirectional change of these parameters [83,86,87,88]. All the particular parameters used for the cumulative indexes, are presented in Supplementary Figures S2–S5.

According to clinical data, patients with epilepsy have increased risk of sudden death, most probably due to the seizure-perturbed heart rhythms characterized by ECG, hence the ECG analysis was employed to estimate the cardiological risks [27,28,29]. ECG was recorded using cutaneous electrodes (disposable silver chloride electrodes for recording ECG in newborns), attached to the right and left on the ventrolateral surface of the animal’s chest. Prior to this, the skin in these areas was degreased with alcohol and the electrodes were glued, fastened to the terminals and mounted on a harness of the original design, which was put on the animal. The connector was linked to a biopotential amplifier having a frequency range of 10 Hz to 20 kHz. The signal from the electrodes was transmitted to an E14-440 analog-to-digital converter (L-Card, Moscow, Russia) connected to a computer. The analog signal was recorded from free-roaming rats using the Powergraph software (DISoft LLC, Moscow, Russia), digitized at a frequency of 1 kHz and processed using Spike-C3, Average and Ints software (OpenScience, Moscow, Russia).

The balance of autonomous regulation of heart rate, representing the relative contribution of the sympathetic and parasympathetic components to the activity of the nervous system, was assessed in accordance with Baevsky et al. [89]. The following parameters of the heart rate variability were calculated: average RR interval in a sample, ms; dX—variance of RR intervals in a sample, ms; parasympathetic, or relaxation, index of the state of the nervous system—RMSSD; sympathetic, or stress, index of the state of the nervous system—SI [89,90].

### 4.6. Preparation of Homogenates of the Rat Cerebral Cortex

Homogenization of the tissue and sonication of homogenates was carried out according to the previously published protocol [67].

### 4.7. Preparation of Tissue Extracts and Quantifications of Metabolites

Methanol-acetate extraction of the rat cerebral cortex and quantification of its amino acids and urea were done as described before [25,91]. Briefly, frozen cerebral cortices were homogenized in ice-cold methanol, then acetic acid solution was added, and the proteins were precipitated. The sodium-citrate buffer system with a Hitachi L-8800 amino acid analyzer was used according to the manufacturer′s User Manual, as described before [25,91].

### 4.8. Measurement of Enzymatic Activities

The activities of thiamine-diphosphate-dependent enzyme complexes of 2-oxoglutarate dehydrogenase and 2-oxoadipate dehydrogenase, as well as the activities of GDH, MDH, GS and ME were measured as previously described [23]. The activity of the pyruvate dehydrogenase complex was determined according to a published protocol for the enzyme assay in tissue homogenates [92]. The activities of aspartate aminotransferase and alanine aminotransferase were measured according to the method of Reitman and Frankel [93]. All of the above activities were assayed in 0.2 mL of the reaction medium spectrophotometrically in transparent 96-well microplates, using a Sunrise plate reader (Tecan, Grödig, Austria).

The activity of PLK was measured using a published assay system with several modifications [94]. First, 2–8 μL of a rat cerebral cortex homogenate with a protein concentration of about 50 mg/mL were added to 0.2 mL of the reaction mixture comprising 50 mM KH_2_PO_4_, pH 6.0; 0.1 mM ZnSO_4_; 0.2 mM pyridoxal; 2 mM Na_2_ATP. The mixture was incubated at 37 °C for 90 min, followed by the cooling of the plate on ice for 5 min and immediate addition of a cold solution of 6 mM hydroxylamine to a final concentration of 1 mM. A control medium without ATP was used as a blank. The fluorescence (ex365 nm | em450 nm) was measured 150 s after the hydroxylamine addition during 5 min. The calculation of the rate of the PLK reaction was carried out as described [94].

Our assay of PNPO was based on the enzymatic production of hydrogen peroxide, which may be detected in complex biological mixtures by Amplex UltraRed (Thermo Fisher Scientific, Carlsbad, CA, USA) [95]. The reaction was carried out at 25 °C in 0.2 mL of the reaction mixture comprising 50 mM HEPES, pH 7.4; 50 mM KCl; 10 mM NaCl; 10 μM pyridoxine 5′-phosphate; 5 U/mL horseradish peroxidase; 10 μM Amplex UltraRed (diluted from 10 mM stock solution in DMSO). To measure the reaction blank, the reaction medium omitting pyridoxine 5′-phosphate was used. The reactions were started with 3–15 μL of the rat cerebral cortex homogenate. The fluorescence (ex560 nm | em590 nm) was measured during the 10 min of the reaction.

The fluorescent assays of PLK and PNPO were done in black microplates using a CLARIOStar plate reader (BMG LABTECH, Ortenberg, Germany).

### 4.9. Statistical Analysis and Data Presentation

Data were analysed using the GraphPad Prism 7.0 software (GraphPad Software Inc., San Diego, CA, USA). Individual values of the parameters for each animal, their median, quartiles and values of the minimum and maximum are shown on the graphs. Statistical significance of differences between the two groups was assessed using the Mann–Whitney U-test. Statistical significance of differences upon comparison of more than two experimental groups was assessed using one-way or two-way analysis of variance (ANOVA) with Tukey’s post hoc test. The statistical significance at *p* ≤ 0.05 is shown on the graphs. The tables below the figures show the ANOVA results for the significance of factors (significant values of *p* ≤ 0.05 are in bold) and their interaction. When mentioned in the text, the ANOVA *p*-values are also supported by the F-values with the degrees of freedom, presented as F(DFn, DFd). In all these cases, the F-values were larger than the corresponding critical values, supporting the significance of the reported results.

## Figures and Tables

**Table 1:** Modified Racine scale for visual assessment of the severity of PTZ-induced seizures in rats [69,70,71].

Score	Behavioral Manifestations of Seizures
0	Normal behavior, no abnormality
1	Immobilization, lying on belly
2	Head nodding, facial, forelimb or hindlimb myoclonus
3	Myoclonic twitches, continuous whole-body myoclonus, tail held up stiffly
4	Rearing, tonic seizure, falling down on a side
5	Tonic-clonic seizure, falling down on back, wild rushing and jumping

**Table 2:** Initial number of animals and their survival in each group.

Animal Sex	Group	*n*	Survival, %
Females	PTZ	9	100
	PTZ +B1,B6 (24 h)	9	100
	PTZ +B1,B6 (2 h)	6	100
	PTZ +B1,B6 (30 min)	5	60
	B1,B6 (24 h)	8	100
	Control	9	100
Males	PTZ	10	100
	PTZ +B1,B6 (24 h)	12	100
	B1,B6 (24 h)	10	100
	Control	9	100

## Data Availability

The data presented in this study are available in this article (summarized in figures and tables, including Supplementary Data). The raw data are available on request from the corresponding author.

## Supplementary Materials

The following are available online at https://www.mdpi.com/article/10.3390/ph14080737/s1, Figure S1: Flowchart of physiological experiments in intact rats (A), rats receiving vitamins B1 and B6 (B), rats receiving PTZ without the vitamins (C), rats receiving PTZ with the vitamins (D), Figure S2: Sex-dependent parameters of behavior 24 h after PTZ-induced seizure, compared to the control animals, Figure S3: Sex-dependent effects of the administration of vitamins B1 and B6 on the behavior 24 h after PTZ-induced seizure, Figure S4: Behavioral effects of administration of vitamins B1 and B6 to the control rats, Figure S5: Dependence of behavioral parameters of the female rats 24 h after the PTZ-induced seizure on the interval between the administration of vitamins B1/B6 and PTZ.

## Abbreviations

PTZ	pentylenetetrazole
OGDHC	2-oxoglutarate dehydrogenase complex
OADHC	2-oxoadipate dehydrogenase complex
PDHC	pyruvate dehydrogenase complex
GDH	glutamate dehydrogenase
MDH	malate dehydrogenase
PLK	pyridoxal kinase
PNPO	pyridoxal 5′-phosphate oxidase;
GS	glutamine synthetase
ME	malic enzyme
GOT	glutamate-oxaloacetate transaminase
GPT	glutamate-pyruvate transaminase
GABA	gamma-aminobutyric acid
