# Supplementary material for: Physiological and Biochemical Markers of the Sex-Specific Sensitivity to Epileptogenic Factors, Delayed Consequences of Seizures and Their Response to Vitamins B1 and B6 in a Rat Model
Source: Pharmaceuticals (Basel). 2021 Jul 28;14(8):737. doi: 10.3390/ph14080737 (PMC8400147; doi:10.3390/ph14080737)
Supplement: Supplementary file 1 [file pharmaceuticals-14-00737-s001.zip › pharmaceuticals-1248682-supplementary.pdf]

Physiological and biochemical markers of the sex-specific sensitivity to epileptogenic factors, delayed consequences of seizures and their response to vitamins B1 and B6 in a rat model

Vasily A. Aleshin, Anastasia V. Graf, Artem V. Artiukhov, Alexandra I. Boyko, Alexander L. Ksenofontov, Maria V. Maslova, Isabel Nogués, Martino L. di Salvo , Victoria I. Bunik

Supplementary Materials

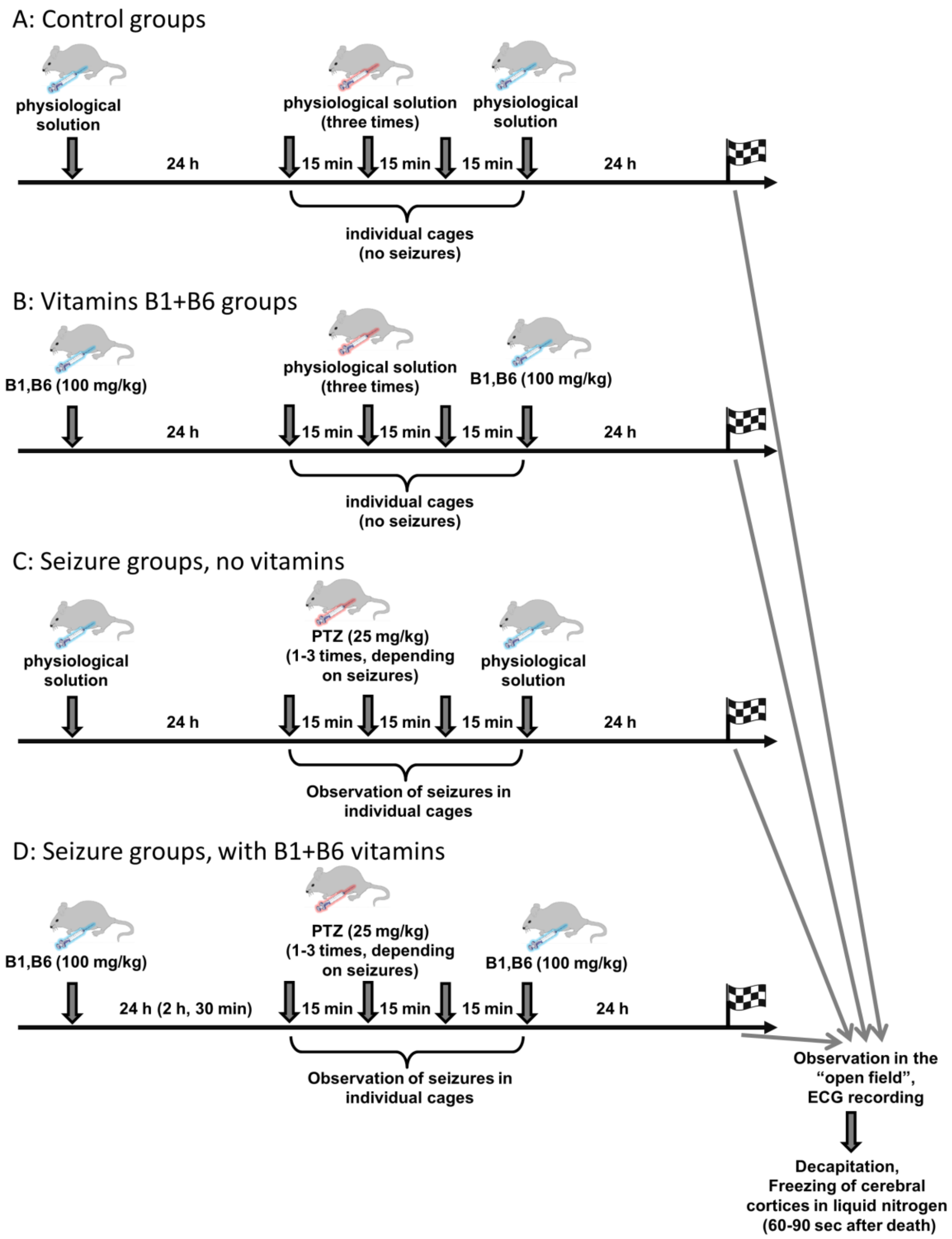

Supplementary Figure S1. Flowchart of physiological experiments in intact rats (A), rats receiving vitamins B1 and B6 (B), rats receiving PTZ without the vitamins (C), rats receiving PTZ with the vitamins (D). The female and male rats received 100 mg/kg of each of thiamine and pyridoxal intraperitoneally 24 h before the first administration of PTZ. The same experimental design was employed in the study of the time dependence of the vitamins effects, which was done on the female rats, where the time of the first administration of the vitamins was 24, 2 or 0.5 h before PTZ **(D)**. PTZ was administered at a dose of 25 mg/kg, followed by estimation of the severity of a seizure during 15 minutes (**Table 1**). In case of a weak seizure (0-3 score points, **Table 1**), PTZ administration and seizure assessment were repeated up to three times. In total, the PTZ dose didn't exceed 75 mg/kg. After 45

minutes of the seizure observation, vitamins B1 and B6 (100 mg/kg each) were injected once again. After 24 hours, the physiological parameters were assessed using an "open field", the ECG was recorded, and the rats were decapitated. The cerebral cortex was quickly removed and frozen in liquid nitrogen (60-90 sec after decapitation). In the control groups the injections of vitamins and/or PTZ were substituted by injections of equivalent volumes of physiological solution (0.9% NaCl).

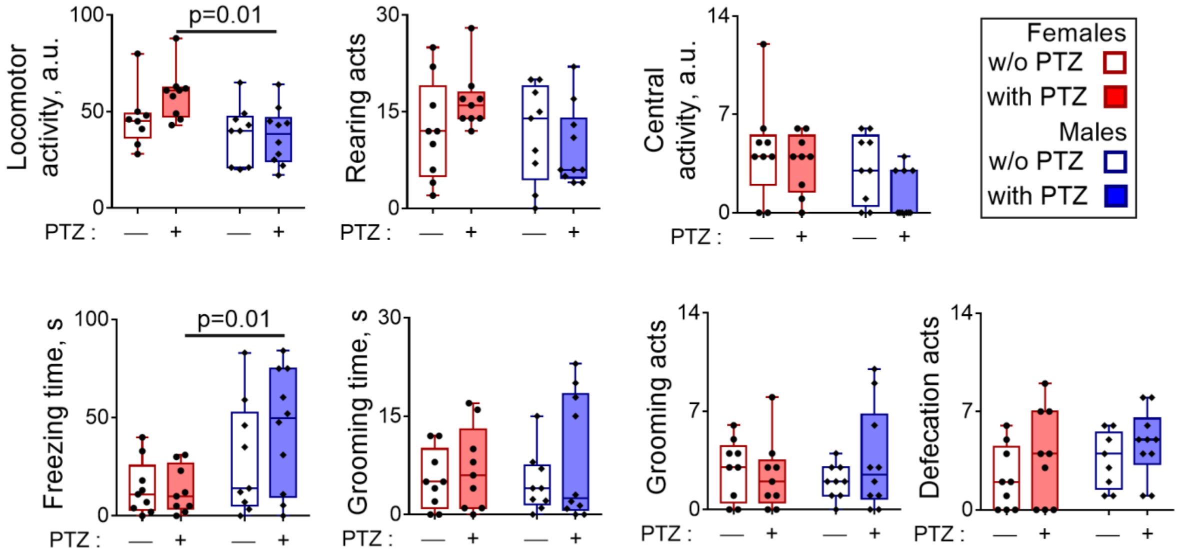

Factor	Locom.	Rearing	Central	Freezing	Gr. time	Gr. acts	Defec.
PTZ:	0.20	0.58	0.10	0.36	0.24	0.54	0.12
Sex:	**<0.01**	0.08	**0.04**	**<0.01**	0.85	0.92	0.19
Interaction:	0.14	0.12	0.54	0.29	0.67	0.25	0.80

Supplementary Figure S2. Sex-dependent parameters of behavior 24h after PTZ-induced seizure, compared to the control animals. The assayed parameters are indicated on the Y axes and represent the ones obtained in the "open field" test (used in Fig. 1 B). P-values are determined using the two-way ANOVA with Tukey's post hoc test (see "Materials and methods").

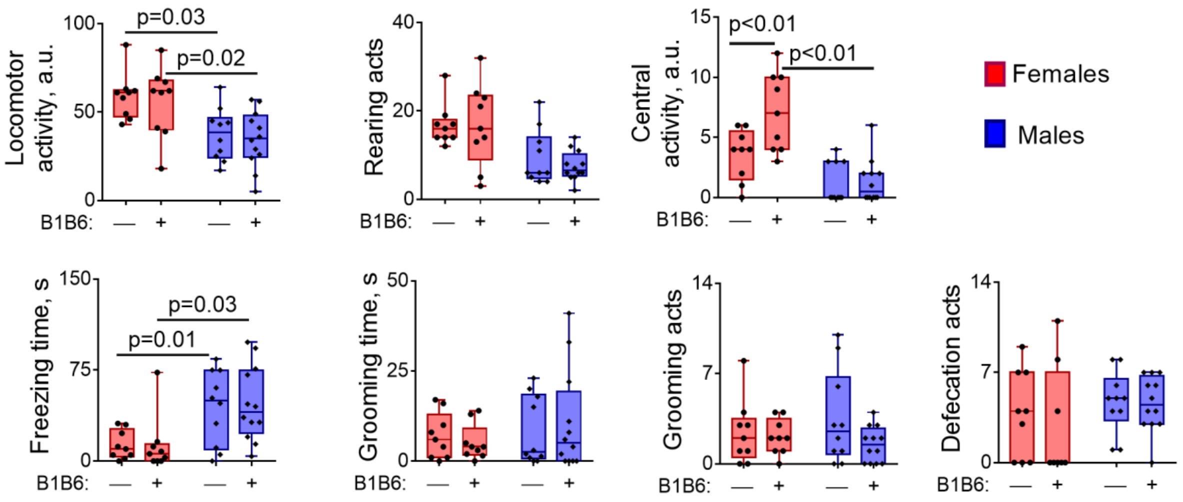

Factor	Locom.	Rearing	Central	Freezing	Gr. time	Gr. acts	Defec.
Vitamins:	0.58	0.63	**0.02**	0.85	0.94	0.13	0.61
Sex:	**<0.01**	**<0.01**	**<0.01**	**<0.01**	0.28	0.77	0.19
Interaction:	0.98	0.63	**0.02**	0.85	0.52	0.28	0.69

Supplementary Figure S3. Sex-dependent effects of the administration of vitamins B1 and B6 on the behavior 24h after PTZ-induced seizure. The assayed parameters are indicated on the Y axes and represent the ones obtained in the “open field” test (used in Fig. 4 B). P-values are determined using the two-way ANOVA with Tukey's post hoc test (see “Materials and methods”).

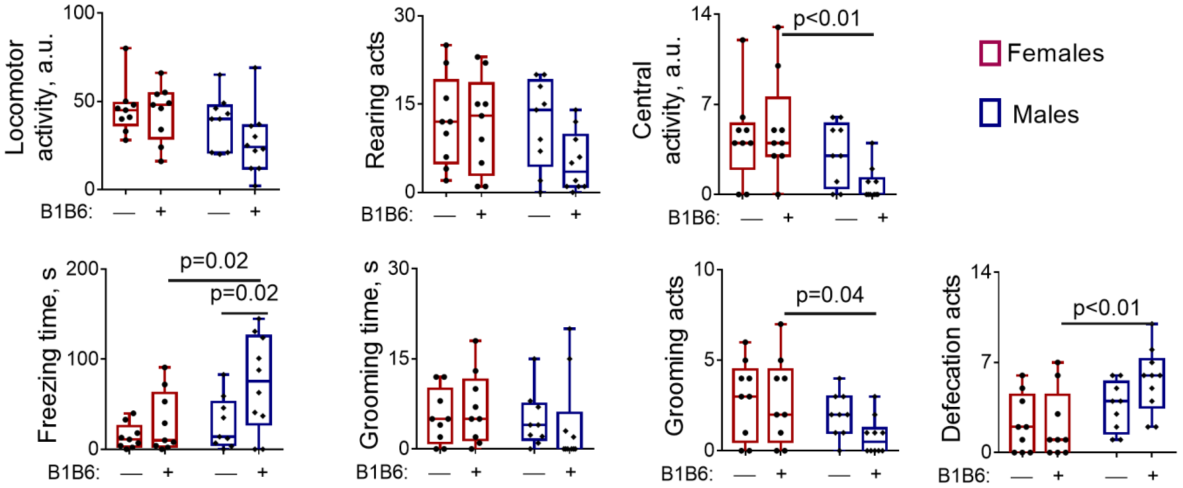

Factor	Locom.	Rearing	Central	Freezing	Gr. time	Gr. acts	Defec.
Vitamins:	0.21	0.14	0.40	**0.02**	0.87	0.27	0.22
Sex:	**0.03**	0.15	**0.04**	**0.02**	0.39	**0.02**	**<0.01**
Interaction:	0.38	0.21	0.10	0.24	0.55	0.36	0.17

Supplementary Figure S4. Behavioral effects of administration of vitamins B1 and B6 to the control rats. The assayed parameters are indicated on the Y axes and represent the ones obtained in the “open field” test (used in Fig. 7). P-values are determined using the two-way ANOVA with Tukey's post hoc test (see “Materials and methods”).

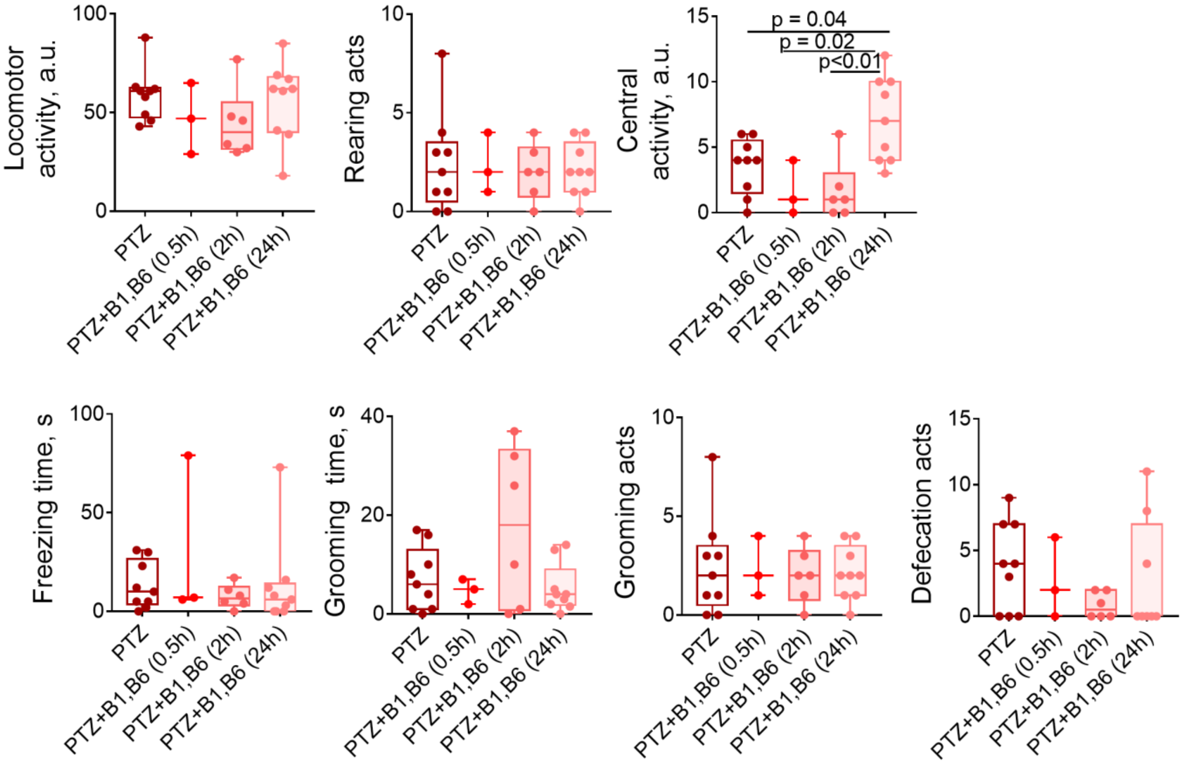

P-values of the time factor:

Locom.	*Rearing*	*Central*	*Freezing*	*Gr. time*	*Gr. acts*	*Defec.*
0.38	0.97	**<0.01**	0.44	0.06	0.09	0.93

Supplementary Figure S5. Dependence of behavioral parameters of the female rats 24h after the PTZ-induced seizure on the interval between the administration of vitamins B1/B6 and PTZ. The assayed parameters are indicated on the Y axes and represent the ones obtained in the “open field” test (used in Fig. 10 B). P-values are determined using one-way ANOVA with Tukey's post hoc test (see “Materials and methods”).
